# Effect of Surface Treatment by O_3_ and Chemical Activation by Alkali Metal on the Performance of ACFs on Adsorption and Desorption of BTX Gases

**DOI:** 10.3390/ijerph17155422

**Published:** 2020-07-28

**Authors:** Jung Hee Jang, Gi Bo Han

**Affiliations:** 1 Department of Applied Environmental Science, Graduate School, Kyung Hee University, Yongin-si, Gyeonggi-do 17104, Korea; azazjh@yahoo.co.kr; 2 Plant Engineering Center, Institute for Advanced Engineering, 633-2 Goan-ri, Baegam-myon, Cheoin-gu, Yongin-si, Gyeonggi-do 17528, Korea

**Keywords:** activated carbon fiber, surface treatment, chemical activation, BTX gas adsorption, textural properties, IAQ

## Abstract

In order to investigate the adsorption characteristics of activated carbon fibers (ACFs) with improved surface morphologies towards volatile organic compounds (VOCs), a commercial low-grade ACF was surface modified by successive surface treatment (ST) and chemical activation (CA) process. O_3_ was used as an ST agent for the formation of oxygen-containing functional groups on the carbon matrix of ACFs. CA was carried out after ST, using a KOH solution. After the successive ST-CA process, Brunauer-Emmett-Teller (BET) surface area and average pore diameter of ACFs were increased from 1483 m^2^/g to 2743 m^2^/g and enlarged from 1.931 nm to 2.512 nm, respectively. The successive ST-CA process also resulted in the adsorption capacities of benzene, toluene, and xylene of the ACFs to increase from 0.22 g_−Ben._/g_−ACFs_, 0.18 g_−Tol._/g_−ACFs_, and 0.19 g_−Xyl_/g_−ACFs_ up to 0.37 g_−Ben._/g_−ACFs_, 0.35 g_−Tol._/g_−ACFs_, and 0.38 g_−Xyl_/g_−ACFs_, respectively.

## 1. Introduction

Volatile organic compounds (VOCs), such as benzene, toluene, and xylene, are air pollutants which are directly related to human health and various environmental problems due to their toxicity. VOCs are emitted during various industrial processes, such as color printing works, paint manufacturing processes, or from gas stations, among others. They can affect human health by affecting the eyes or the nose and could cause cancer when people are exposed to them for a long time, even at low concentrations [1,2,3]. Due to the growing awareness of the general public about the influence of air quality on health and wellbeing, these issues have emerged as subjects of regulatory interest world-wide [4,5].

Emission of VOCs is controlled by stringent environmental regulations, and removal processes have been developed for the efficient control of their presence in the atmosphere. Currently developed technologies, such as thermal incineration and wet scrubbing, have weak points which are usually their high cost and low efficiency, plus their applicability in the especial cases and areas when VOCs are emitted at low concentrations. The efficient technologies to treat gaseous VOCs are the following: absorption, adsorption, condensation, thermal oxidation, catalytic oxidation, and photocatalytic oxidation [6,7,8,9,10,11]. These technologies show high efficiency and effectiveness towards VOC treatment when conditions such as flow rate, temperature, and VOC concentration are appropriately optimized. In order, however, to obtain high performance and technology optimization, rigorous research needs to be conducted.

Activated carbon fibers (ACFs), among various carbon-based materials, have been used for the adsorption and removal of gases and odors from the air for a long time, owing to their homogeneous pore structure and large BET (Brunauer-Emmett-Teller) surface area [10]. ACFs have a particular type of structure and show rapid adsorption kinetics compared with powder- and granule-type activated carbons. They can also be used for special purposes because they have physical forms that can be processed in various forms, such as felt and cloth [12]. Preparation methods of porous, carbon-based materials are conventionally classified into physical and chemical activation. Chemical activation of ACFs preparation is usually carried out via a heat treatment process of the ACF precursors, in presence of a chemical additives, such as potassium, sodium, phosphorous, and magnesium [13,14]. Recently, potassium hydroxide has been used as a chemical activation agent for porous polymers [15,16,17,18,19]. 

The thin-fiber shape with short and straight micropore mean ACFs have faster intraparticle adsorption kinetics than Activated carbon (AC). ACFs are easier to handle into desired forms such as felt or fabric, which is convenient for engineering applications. Therefore, ACFs are a good candidate for gas adsorption for their large adsorption capacity and high mass transfer rates during the adsorption or desorption process. The development of high-surface ACFs, while maintaining the ACF characteristics, allows high-performance small air purification facilities to be built [20,21,22,23].

In the present study, commercial, rayon-based ACFs were successively surface-treated and chemically activated to improve morphological characteristics, such as porosity and surface area. The objective of this study was to develop a modified ACF through a successive surface treatment (chemical activation process) and apply them for benzene, toluene, and xylene (BTX) gas adsorption. Furthermore, the effect of the porosity and surface area on the BTX gas adsorption capacity of ACFs and their life time was investigated using a cyclic adsorption–desorption process. Thus, the development and applicability of highly efficient ACFs for the BTX gas adsorption process was discussed for the removal, separation, and recovery of BTX gases.

## 2. Materials and Methods

### 2.1. Successive ST-CA Process for the Preparation of the Upgraded ACFs

**Figure 1:**
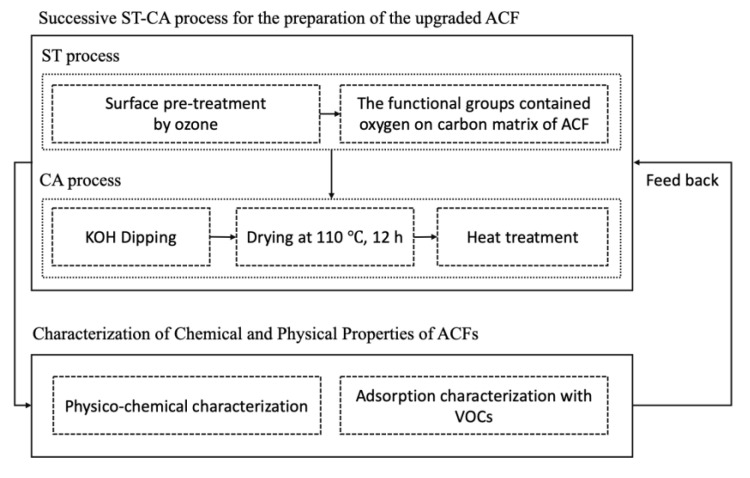
Proposed framework for Successive ST-CA (surface treatment-chemical activation) process for the preparation of the modified ACFs (Activated carbon fibers). Volatile organic compounds (VOCs).

The three stages of this study are depicted in Figure 1. The first stage is the surface treatment (ST) process using ozone (O_3_), the second stage is the chemical activation (CA) using alkali metal, and the third stage is the evaluation of surface properties for the modified ACFs. Rayon of low grade, commercially available in China, was used as the ACF precursor, to develop the upgraded ACFs in this study.

**Figure 2:**
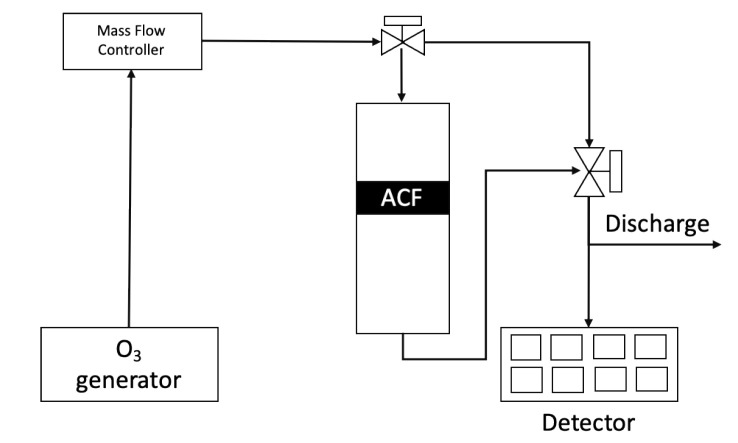
Reactor process flow diagram for surface treatment using ozone.

Figure 2 shows the reactor used for surface treatment using O_3_. The surface treatment of as-received commercial ACFs was conducted with O_3_ at room temperature, followed by a Pyrex tube used as reactor. O_3_ used for the surface treatment was generated from an industrial O_3_ generator and fed into the reactor packed with ACFs. The feed O_3_ concentration was fixed at 20 g/m^3^ and the outlet concentration of O_3_ emitted from the reactor was detected with an O_3_ analyzer(OZM-7000, Ozonetech, South Korea). The flow rate of O_3_ balanced by O_2_ was about 200 mL/min and the mass of the packed ACFs was about 0.8 g. The temperature of the packed ACFs layer was also monitored.

The chemical activation of the surface-treated ACFs was carried out using KOH as follows. A total of 1.0 g of O_3_-treated ACFs was soaked in 1 M KOH solution and aged overnight in an ultrasonicator. The KOH-impregnated ACFs were separated from 1 M KOH solution and were dried at 117 °C for 12 h, in an electronic drying oven. The dried ACFs were placed in a horizontal quartz tube reactor and heat-treated at 800 °C for 2 h under Ar gas flow (500 mL/min). To remove the potassium, the heated-treated ACFs were rinsed with 250 mL of 1 M H_2_SO_4_ solution and were washed and filtered with distilled water until the pH of filtrate reached about 6. The washed ACFs were dried at 110 °C overnight.

### 2.2. Characterization of Chemical and Physical Properties of ACFs

Elemental analysis of ACFs was conducted to investigate the elemental composition of ACFs before and after the surface treatment and chemical activation process. Also, Fourier Transform Infrared Spectroscopy (FT-IR) were measured on a Germany Bruker IFS 28CS spectrometer with deuterated triglycine sulfate(DTGS) detector and OPUS/IR 2.0 software. FT-IR analysis was conducted to find out the formation and decomposition of the oxygen-contained functional groups, such as carbonyl and carboxyl groups during surface treatment and chemical activation.

A Micromeritics ASAP 2010 volumetric adsorption analyzer (USA) was used to measure Ar (99.999% pure) adsorption–desorption isotherms at −186 °C, in the 10–6–1 relative pressure range. The apparatus was equipped with 1000, 100, and 1 Torr transducers, allowing for high accuracy and resolution of the measurements. Prior to the measurements, all samples were degassed overnight at 250 °C under vacuum (2.7 × 10^−3^ kPa). The repeatability of the isotherms between different runs was better than 1.5%. The cross-sectional area employed for Ar adsorption at −186 °C was 0.154 nm^2^. The surface area of the samples was assessed by the standard BET method, using Ar adsorption data in the relative pressure range from 0.01 to 0.10, since deviations of the BET plots from linearity were observed for relative pressure <0.01 and >0.10. The total pore volume was calculated by converting the amount of Ar adsorbed at a relative pressure of 0.975 to the volume of liquid adsorbate. Pore volume and pore diameter were calculated using the H-K computation method (Horvath and Kawazoe), estimating slit-pore geometry.

### 2.3. VOCs Adsorption—Desorption with ACFs before and after Surface Treatment and Chemical Activation

**Figure 3:**
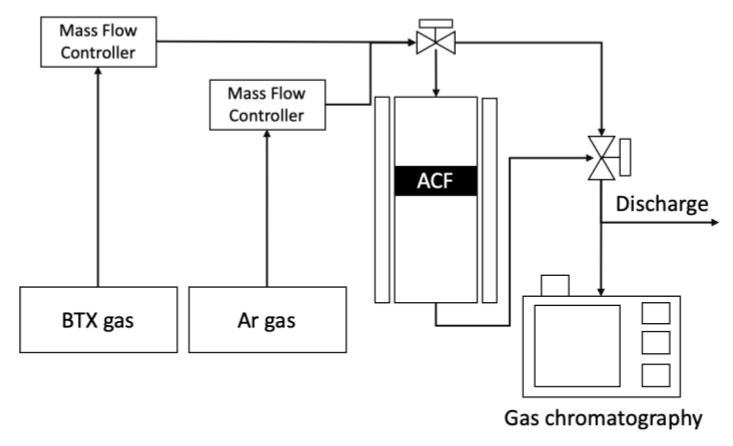
Process flow diagram of the VOCs adsorption–desorption system. BTX: benzene, toluene, and xylene.

The reactor used for BTX gas adsorption–desorption experiments is shown in Figure 3. Adsorption of VOCs was performed using a laboratory scale unit to evaluate the adsorption capacity of the prepared ACFs towards benzene, toluene, and xylene. The amount of ACFs packed in a quartz-tube reactor with outer diameter(OD) of 1/2 inch and length of 40 cm, before and after the ST-CA process, was 0.05 g. Adsorption was carried out under a flow rate of 300 mL/min and a concentration of 1000 ppmv (balanced by N_2_) at 30 °C. Once adsorption was completed, desorption was conducted by flowing Ar gas of 300 mL/min, at 200 °C. The inlet and outlet concentrations of VOCs passed through the fixed ACFs reactor bed were analyzed by gas chromatography, equipped with an alumina capillary column and flame ionization detector (FID), in order to define the breakthrough curve for VOCs. The adsorption capacity of ACFs towards VOCs was calculated by the integration of VOC concentration until the adsorption amount was saturated. After the adsorption step was conducted by confirming the saturated adsorption of toluene, desorption to regenerate ACFs was carried out by the thermal treatment under Ar atmosphere.

## 3. Results and Discussion

**Figure 4:**
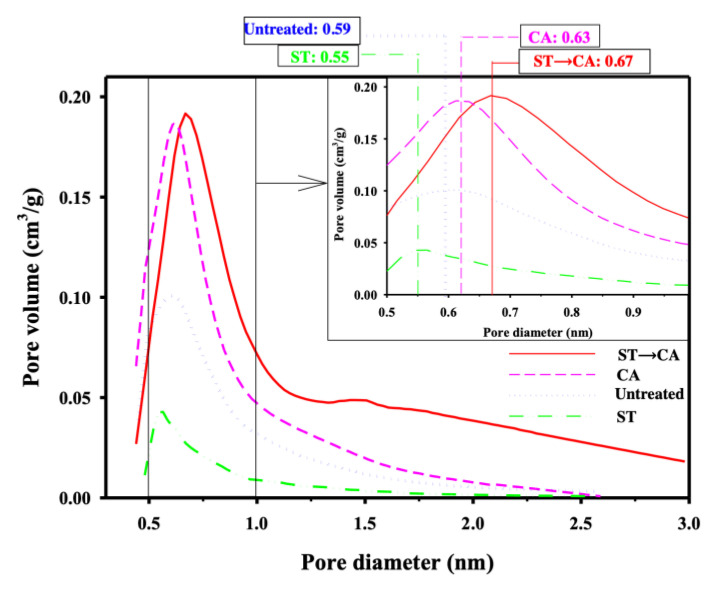
Pore size distribution of ACFs before and after surface treatment and chemical activation (Untreated, untreated ACFs; ST, ACFs prepared through surface treatment; CA, ACFs prepared through chemical activation without prior surface treatment; ST→CA, ACFs prepared through chemical activation after surface treatment).

Figure 4 shows the pore size distribution curves of ACFs before and after the surface treatment and chemical activation. The peak corresponding to maximum pore volume was located at 0.59 nm and most of the pore volume was distributed in the pore range below 2.5 nm. The total pore volume of ACFs prepared through chemical activation, without prior surface treatment, was larger than that of the untreated ACFs, and the peak corresponding to maximum pore volume was located at 0.63 nm. This indicates that the pore structure and pore size were increased during the chemical activation without prior surface treatment compared to the untreated ACFs. The pore size of ACFs undergoing surface treatment was distributed in the range below 2.0 nm, and its maximum peak was shifted from 0.59 to 0.55 nm compared to the untreated ACFs. Also, the total pore volume of ACFs after surface treatment was smaller than that of the untreated ACFs. These results can be explained due to the formation of oxygen-containing functional groups on ACFs and the reduction of the pore volume with the surface treatment.

The pore volume of ACFs prepared through chemical activation following the surface treatment was enlarged, and the peak corresponding to maximum pore volume was located at 0.67 nm, larger than ACFs before and after chemical activation without surface treatment. These results can be explained based on the fact that the pore volume and size of ACFs prepared through chemical activation conducted after surface treatment were increased because the oxygen-containing functional groups formed during the surface treatment were decomposed and destroyed during the chemical activation. Therefore, it was shown that surface treatment followed by chemical activation can be proposed as a method to prepare ACFs with enlarged pore volume and pore size.

**Figure 5:**
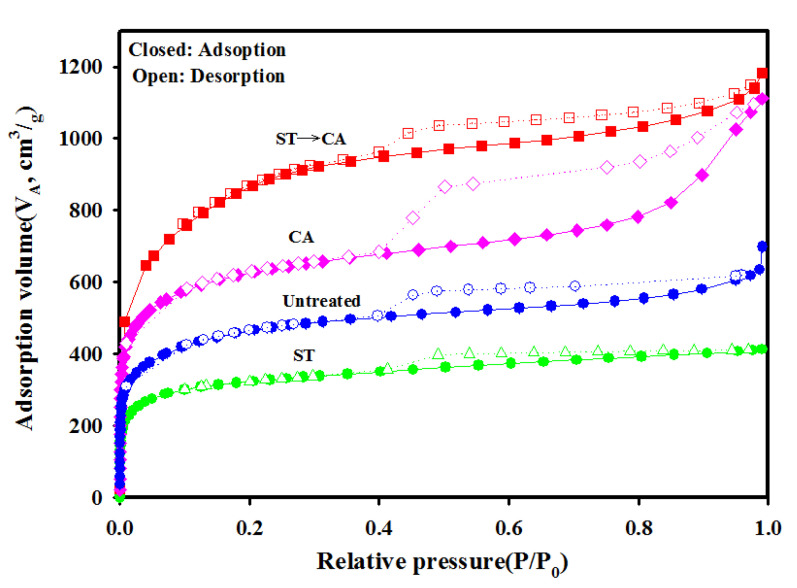
Isothermal Ar adsorption–desorption curves of ACFs (Untreated, untreated ACFs; ST, ACFs prepared though surface treatment for 80 min; CA, ACFs prepared through chemical activation without prior surface treatment; ST→CA, ACFs prepared through chemical activation after surface treatment for 80 min).

Figure 5 shows the isothermal Ar adsorption–desorption curves of ACFs under various treatment combinations. The morphological properties of ACFs obtained before and after the surface treatment and chemical activation were investigated and calculated using the isothermal curves. Isotherms obtained for ACFs are similar to the theoretical type I(b) curve, characteristic for microporous materials with pore size distributions over a broad range including wide microporosity (0.7–2.0 nm) and narrow mesoporosity (>2.5 nm) [24]. The typical feature of type I(b) curve is the long plateau extending over a very wide range of relative pressure (P/P0).

Table 1 reports morphological parameters calculated from the isothermal Ar adsorption curves at −185.71 °C. The untreated ACFs showed a BET surface area of 1483 m^2^/g, average pore diameter of 1.931 nm, average micropore diameter of 0.744 nm, and micropore area of 1199 m^2^/g. The BET surface area and pore volume obtained for ACFs prepared through chemical activation without prior surface treatment were about 1998 m^2^/g and 1.414 cm^3^/g, respectively, higher than the values for untreated ACFs. Also, the pore size of chemically activated ACFs was enlarged compared to untreated ACFs. After ACFs underwent surface treatment, the pore size, pore volume, and BET surface area were reduced from 1.931 nm, 0.7512 cm^3^/g, and 1483 m^2^/g to 1.841 nm, 0.4313 cm^3^/g, and 796 m^2^/g, respectively. This change was attributed to the pore structure being blocked by the oxygen-containing functional groups formed during the surface treatment. It was estimated that the pore structure remained the same after surface treatment, because the texture properties of the pore size, volume, and BET surface area were measured similar to the untreated ACFs after the heat temperature at 800 °C, as shown in Table 1. The ACFs prepared through chemical activation after surface treatment took place show 2743 m^2^/g for BET surface area, 1.5013 cm^3^/g for total pore volume, 2.512 nm for average pore diameter, 1.014 nm for average micropore diameter, and 0.9621 cm^3^/g for micropore volume, values higher than all other ACFs samples examined. This result may be attributed to the fact that the pore structure was developed and enlarged by chemical activation conducted after surface treatment, because of a synergistic effect. The oxygen-containing functional groups, which act as defect sites and seeds to develop the pore structure, were formed over the carbon matrix of ACFs during the surface treatment and were then decomposed and destroyed during the chemical activation.

**Figure 6:**
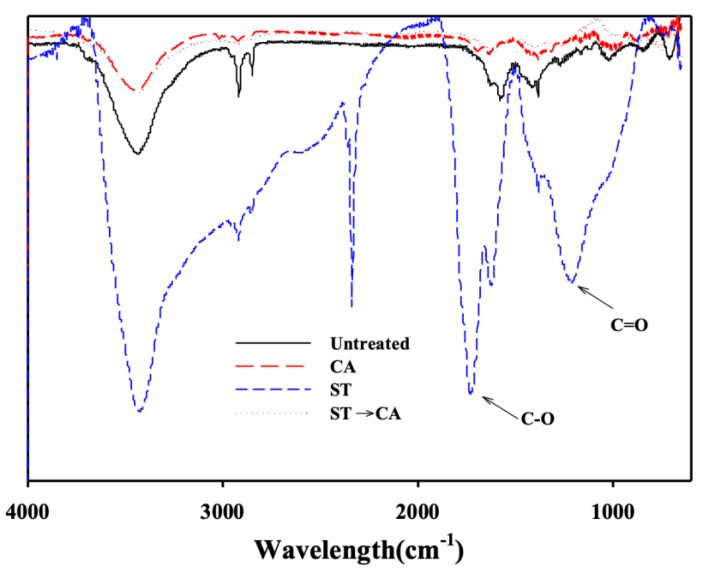
FT-IR (Fourier Transform Infrared Spectroscopy) results of ACFs ST-CA process (Untreated, untreated ACFs; ST, ACFs prepared through surface treatment; CA, ACFs prepared through chemical activation without prior surface treatment; ST→CA, ACFs prepared through chemical activation after surface treatment).

Figure 6 shows the FT-IR spectra obtained for ACFs samples through various treatment combinations. According to literature, oxygen-containing functional groups are formed on the surface of carbon surface treated by ozone [25]. Obtained FT-IR spectra show that the band corresponding to the C=O functional group can be observed at 1750 cm^−1^ and 1860 cm^−1^, and the C–O band can be seen at 1190 cm^−1^ [26,27]. Those peaks were not observed on the FT-IR spectra for untreated ACFs. The surface-treated ACFs showed increased peaks around the wavelength of 1750 and 1190 cm^−1^. This is attributed to the fact that the oxygen present on the ACFs surface and the oxygen supplied by O_3_ were combined to form an oxygen-containing functional group. When the surface-treated ACFs were chemically activated, the until then present peak of the ACFs oxygen functional group disappeared. This is attributed to the thermal decomposition of the oxygen-containing functional groups on the ACFs surface during the heat treatment process included in the chemical activation.

During the heat treatment process included in the chemical activation using KOH, carbon on the ACFs surface is oxidized to CO_2_ as shown in the reaction in Formula (1). After conversion to CO_2_, the area around the “lost” carbon develops into a pore [28,29].

4KOH + C ↔ 4K + CO_2_ + 2H_2_O (1)

**Figure 7:**
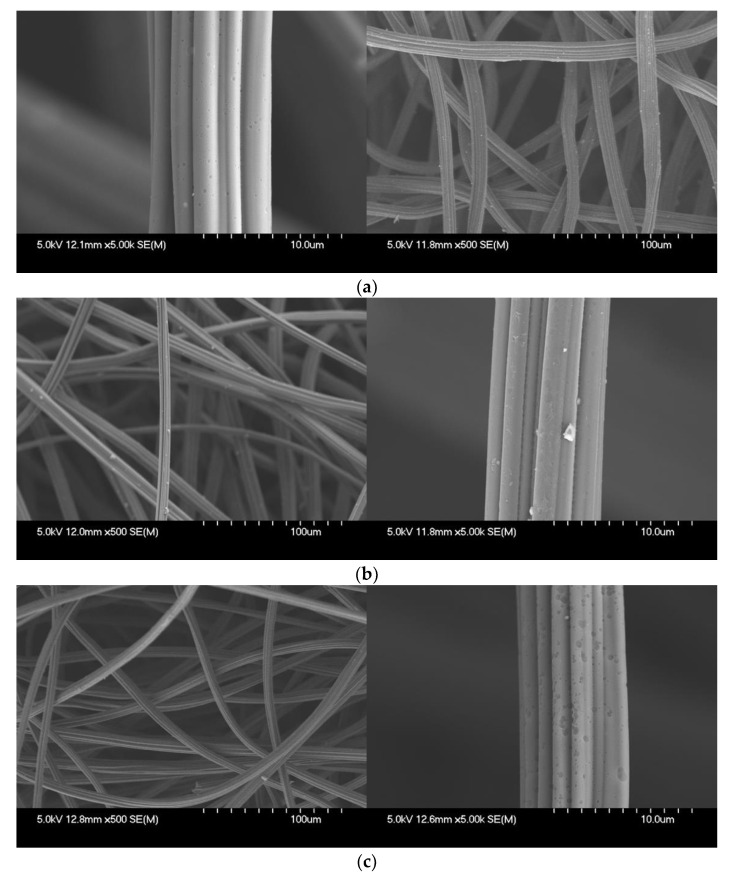
Results of surface morphology of ACFs after ST-CA process obtained via Scanning Electron Microscope (SEM). (**a**) Untreated ACFs, (**b**) ACFs after ST process, (**c**) ACFs after ST-CA process.

Figure 7 shows the results of surface morphology of ACFs after the ST-CA process. The observation result in a magnification of ×500 shows that the diameter of the ACFs was 11 µm on average, with no significant change before and after the ST-CA process. Observing the structure of the ACFs under a magnification of ×5000, shows that the surface of ACFs became rough after surface treatment and chemical modification. This can be seen as a result of pore development on ACFs, due to loss of carbon components through the generation and disappearance of oxygen-containing functional groups, during activation of the carbon surface.

**Figure 8:**
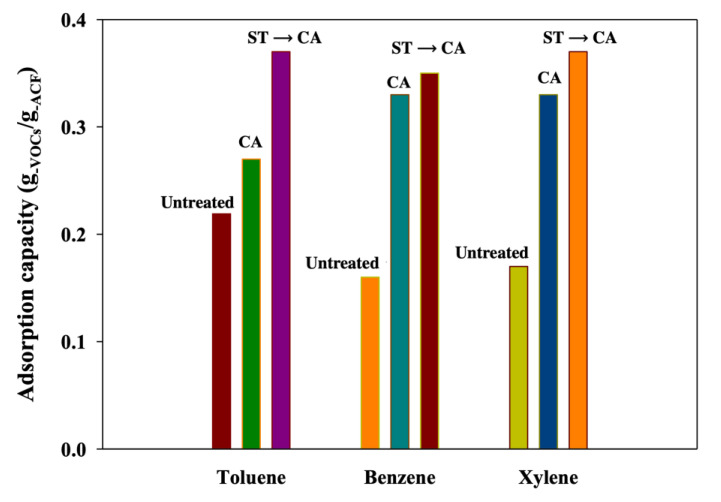
Adsorption capacity in BTX gas adsorption process with ACFs (Untreated, untreated ACFs; ST, ACFs prepared through surface treatment; CA, ACFs prepared through chemical activation without prior surface treatment; ST→CA, ACFs prepared through chemical activation after surface treatment).

Based on the improved morphological properties of ACFs after the successive ST-CA process, the adsorption characteristics of benzene, toluene, and xylene for the untreated and modified ACFs were examined. Figure 8 shows the adsorption capacity of the untreated and modified ACFs for benzene, toluene, and xylene. The toluene adsorption capacity of ACFs was raised from 0.22 to 0.27 g_−Tol._/g_−ACFs_ after CA process and up to 0.37 g_−Tol._/g_−ACFs_ after ST-CA process. The adsorption capacity for benzene was improved from 0.16 to 0.33 g_−Ben._/g_−ACFs_ after CA process and increased to 0.35 g_−Ben._/g_-ACFs_ after the combined ST-CA process. The adsorption capacity for xylene increased from 0.17 to 0.33 g_−Xyl._/g_−ACFs_ after the CA process and further to 0.37 g_−Xyl._/g_−ACFs_ after the ST-CA process. It was assumed that the adsorption capacity of ACFs for VOCs was improved after the CA and ST-CA processes, because the adsorption capacity of toluene, benzene, and xylene was proportionally influenced by the BET surface area and pore structure, which were simultaneously expanded and improved by the pore development through the CA and ST-CA processes. In general, morphological properties, such as BET surface area, pore structure, and VOCs adsorption capacity, were improved more by the ST-CA process, than the CA process alone. Pore development was affected more drastically from the ST process rather than the CA process.

**Figure 9:**
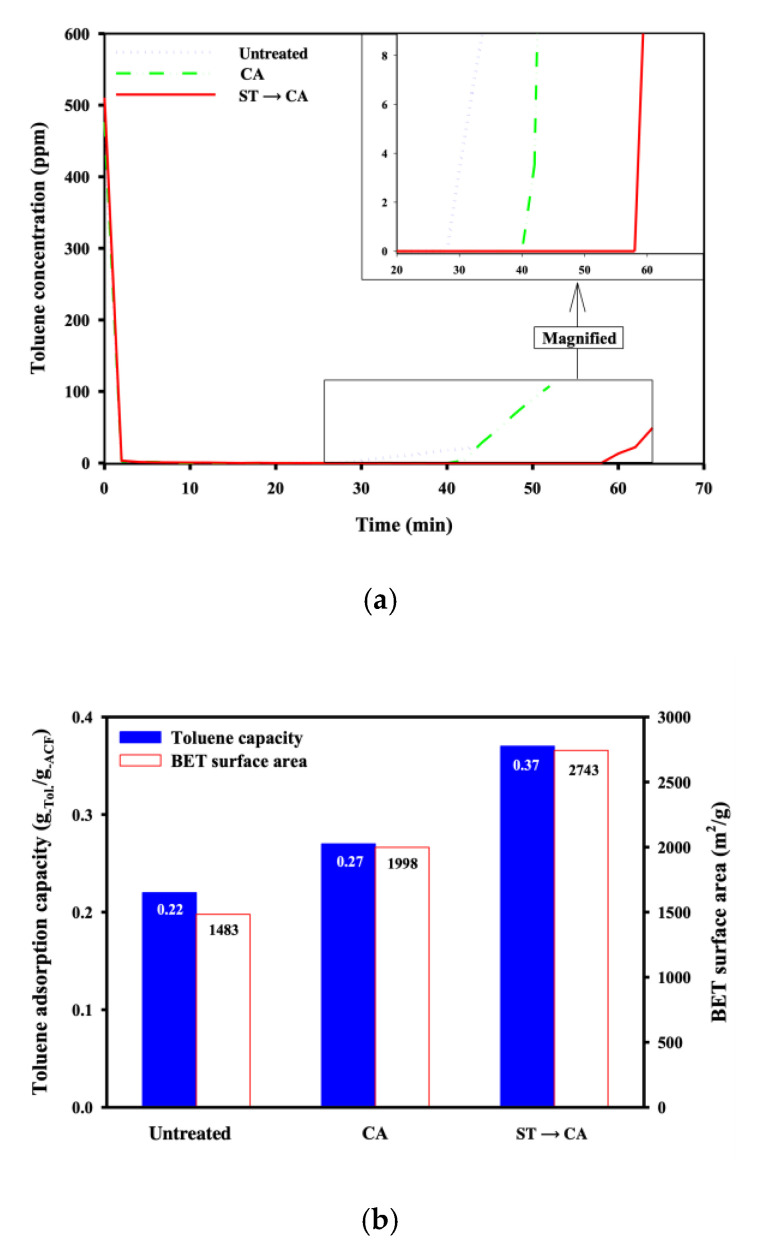
(**a**) Breakthrough curves and (**b**) adsorption capacity of ACFs for toluene and BET surface areas of ACFs (Untreated, untreated ACFs; ST, ACFs prepared in the surface treatment for 80 min; CA, ACFs prepared in the chemical activation without the surface treatment; ST→CA, ACFs prepared in the chemical activation after the surface treatment for 80 min).

In order to find out the relationship between the morphological properties and the performance of ACFs, toluene adsorption was carried out using ACFs obtained before and after sequential surface treatment and chemical activation processes. Figure 9 shows the breakthrough curves and toluene adsorption capacities. As shown in Figure 9, the breakthrough point time for the untreated ACFs was about 27 min and the measured toluene adsorption capacity was about 0.22 g_−Tol._/g_−ACFs_. The ACFs prepared through chemical activation, without prior surface treatment showed a breakthrough point time of about 40 min, and the toluene adsorption capacity measured was 0.27 g_−Tol._/g_-ACFs_, both increased compared to those for untreated ACFs. It was assumed that the increase of the toluene adsorption capacity resulted from the improvement of the morphological properties due to the chemical activation without prior surface treatment. The breakthrough point time and toluene adsorption capacity for ACFs prepared through chemical activation conducted after surface treatment were about 58 min and 0.37 g_−Tol._/g_-ACFs_, respectively, and they were the highest values among the examined ACFs before and after surface treatment and chemical activation processes. Comparing the values obtained for all ACFs examined, before and after treatments, showed that the breakthrough point time and toluene adsorption capacity were proportional to the BET surface area. Hence, it was estimated that this performance superiority of ACFs prepared through chemical activation conducted after surface treatment is attributed to the superior morphological properties obtained through the synergistic effect between the formation of the oxygen-containing functional groups and the development of the pore structure through the combination of treatments.

## 4. Conclusions

Activated Carbon Fibers (ACFs) with high surface area and improved surface morphologies were prepared by a successive process of surface treatment by O_3_ and chemical activation by alkali metal. The effect of the improved physical and chemical properties of ACFs on the adsorption of volatile organic compounds (VOCs), such as benzene, toluene, and xylene, was investigated. The morphological characteristics of ACFs, such as BET surface area, pore size, and pore volume, were improved by the synergistic effect between the formation of oxygen-containing functional groups with surface treatment and the development of the pore structure by chemical activation. Existing pores were expanded, and new pores were developed. This morphological improvement resulted in the improvement of the adsorption capacity of the prepared ACFs towards toluene, benzene, and xylene.

Also, the BTX adsorption performance test confirmed that the adsorption capacity increased with an increase in BET after the surface treatment and refinement process. Our results suggest that VOCs present in interior spaces can be effectively removed by utilizing structurally improved ACFs.

## Figures and Tables

**Table 1:** Textural properties of ACFs (Untreated, untreated ACFs; ST, ACFs prepared through surface treatment for 80 min; CA, ACFs prepared through chemical activation without prior surface treatment; ST→CA, ACFs prepared through chemical activation after surface treatment for 80 min; *n* = 7).

Textural Properties of ACFs	Untreated	ST		CA	ST→CA
		Before Heat Treatment	After Heat Treatment		
BET surface area (m^2^/g)	1483 ± 128.2	796 ± 76.3	1390 ± 108.2	1998 ± 140.2	2743 ± 203
Total pore volume (cm^3^/g, P/Po = 0.99)	0.7512 ± 0.063	0.4313 ± 0.045	0.5498 ± 0.044	1.414 ± 0.141	1.5053 ± 0.193
Average pore diameter (nm)	1.931 ± 0.082	1.841 ± 0.077	1.959 ± 0.074	1.912 ± 0.072	2.512 ± 0.12
Average micropore diameter (nm)	0.744 ± 0.065	0.715 ± 0.080	0.759 ± 0.044	0.735 ± 0.054	1.014 ± 0.015
Micropore volume (cm^3^/g, P/Po = 0.1)	0.5477 ± 0.034	0.2929 ± 0.017	0.4992 ± 0.023	0.7346 ± 0.045	0.9621 ± 0.075
Micropore volume percent (%)	72.9 ± 5.66	67.9 ± 4.31	88.3 ± 2.4	51.9 ± 10.2	63.9 ± 3.14
Micropore area (m^2^/g, P/Po = 0.1)	1199 ± 102.3	398 ± 20.9	1227 ± 10.3	2099 ± 180.2	2331 ± 222.1
External area (m^2^/g, P/Po = 0.1)	165 ± 15.6	30 ± 3.2	163 ± 10.6	136 ± 20.3	310 ± 29.2

ST: surface treatment, CA: chemical activation, BET: Brunauer-Emmett-Teller.
